# Polycystic Ovary Syndrome and Ferroptosis: Following Ariadne’s Thread

**DOI:** 10.3390/biomedicines12102280

**Published:** 2024-10-08

**Authors:** Styliani Geronikolou, Athanasia Pavlopoulou, Ioannis Koutelekos, Dimitrios Kalogirou, Flora Bacopoulou, Dennis V. Cokkinos

**Affiliations:** 1 Clinical, Translational and Experimental Surgery Research Center, Biomedical Research Foundation of the Academy of Athens, 11527 Athens, Greece; dcokkinos@bioacademy.gr; 2 Center for Adolescent Medicine and UNESCO Chair in Adolescent Health Care, First Department of Pediatrics, School of Medicine, National and Kapodistrian University of Athens, 11527 Athens, Greece; fbacopoulou@med.uoa.gr; 3 Izmir Biomedicine and Genome Center (IBG), 35340 Izmir, Turkey; athanasia.pavlopoulou@deu.edu.tr; 4 Izmir International Biomedicine and Genome Institute, Dokuz Eylül University, 35340 Izmir, Turkey; 5 Department of Nursing, School of Health and Care Sciences, University of West Attica, 12243 Athens, Greece; ikoutel@uniwa.gr; 6 Department of Public and Community Health, School of Public Health, University of West Attica, 11521 Athens, Greece; kalogirou2005@gmail.com

**Keywords:** ferroptosis, PCOS, interactions network, systems medicine, genetic epidemiology, polycystic ovary syndrome, genes

## Abstract

**Background:** Recent literature suggests that ferroptosis (FPT) may be a key player in polycystic ovary syndrome (PCOS) pathogenesis, but the underlying mechanism(s) remain(s) unclear. **Aim:** Therefore, herein, we made an effort to reproduce the molecular signature of the syndrome by including FPT and exploring novel drug targets for PCOS. **Methods:** (a) Our previously constructed PCOS interactions molecular network was extended with the addition of FPT–associated genes (interaction score above 0.7) and (b) gene set enrichment analysis was performed so as to detect over-represented KEGG pathways. **Results:** The updated interactome includes 140 molecules, 20 of which are predicted/novel, with an interaction score of 7.3, and 12 major hubs. Moreover, we identified 16 over-represented KEGG pathways, with FPT being the most overexpressed pathway. The FPT subnetwork is connected with the PCOS network through KDM1A. **Conclusions:** FPT cell death is involved in PCOS development, as its major hub TP53 was shown to be the most important hub in the whole PCOS interactome, hence representing a prioritized drug target.

## 1. Introduction

Polycystic ovary syndrome (PCOS) is one of the chronic composites and difficult-to-treat disorders with genetic, epigenetic, and environmental facets. Its clinical image is compound, involving hirsutism, uneven menstruation, acne, and occasionally, infertility [1]. Four clinical phenotypes have been suggested by the Rotterdam criteria with regard to the clinical, metabolic, and hormonal profile (Table 1) [2,3]. Its prevalence depends on the diagnostic tools adopted, varying from 6 to 20% [4]. The geographic and ethnicity variation has been attributed to the different diagnostic criteria adopted, but the recent literature raises concerns about racial, genetic, and cultural implications [5]. Its treatment is directed towards relieving distressing symptoms, including changes in lifestyle, weight loss, and menstrual cycle regulation by administrating contraceptives and/or anti-diabetic drugs [6]. In a previous work of ours, we had revealed that metabolic disturbances lie upstream from the reproductive ones, linking together through insulin [6].

Iron metabolism has been suggested as a contributing factor in endocrine pathology including PCOS [7,8]. Ferroptosis (FPT) is a distinct form of programmed cell death, originating from the iron-dependent aggregation of lipid hydroperoxides [9,10].

The mechanism involved especially in PCOS pathology is poorly understood, thus meriting further research. So far, in silico and in vivo investigations have generated limited information. Animal models and human studies have focused on illuminating the phenomenon; however, this target is both costly and time-consuming. We opted to study the mechanism in silico so as to save time and costs, and direct further in vivo research targets.

## 2. Materials and Methods

**Figure 1:**
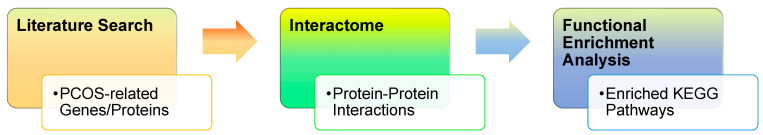
Flow chart describing the overall methodology followed herewith.

The overall methodology followed in this study is illustrated in Figure 1.

### 2.1. Molecular Network

PCOS-related and FPT–PCOS-associated genes/proteins were retrieved through an extensive literature search of the bibliographic database PubMed/MEDLINE up to the current date. Given that the proteins implicated in the same disease tend to interact, either physically or functionally, with each other [11], the interactions among these gene products were investigated through STRING v12.0 [12], a database of both known and predicted associations among genes/proteins. A high confidence interaction score of above 0.7 was chosen. To avoid false positives (i.e., erroneous associations), only those interactions derived from experiments, text mining of published studies, and curated knowledge bases of protein complexes and pathways were included. Moreover, genes/gene products not previously reported to be associated with PCOS or FPT–PCOS, and therefore not included in the initial set of molecules in STRING, are referred to as “novel” throughout the manuscript; the latter were identified through iterative searches for the minimum number of nodes interacting with the existing nodes.

### 2.2. Functional Enrichment Analysis

To interpret in a biologically meaningful way the components of the PCOS interactome, gene set enrichment analysis (GSEA) was performed to identify relevant statistically significant KEGG (Kyoto Encyclopedia of Genes and Genomes) [13] pathways over-represented in those genes coding for the proteins that comprise the PCOS interaction network. GSEA was conducted with WebGestalt (WEB-based GEne SeT AnaLysis Toolkit) 2024 [14], an online tool used for the identification of significantly enriched terms in given gene sets. The default advanced parameters were selected, and the KEGG pathways with false discovery rate (FDR)-adjusted *p*-value less than 0.05 were considered in the analysis. The weighted set cover algorithm was used for clustering the terms by selecting a subset of representative terms.

## 3. Results

### 3.1. Molecular Network Construction

**Figure 2:**
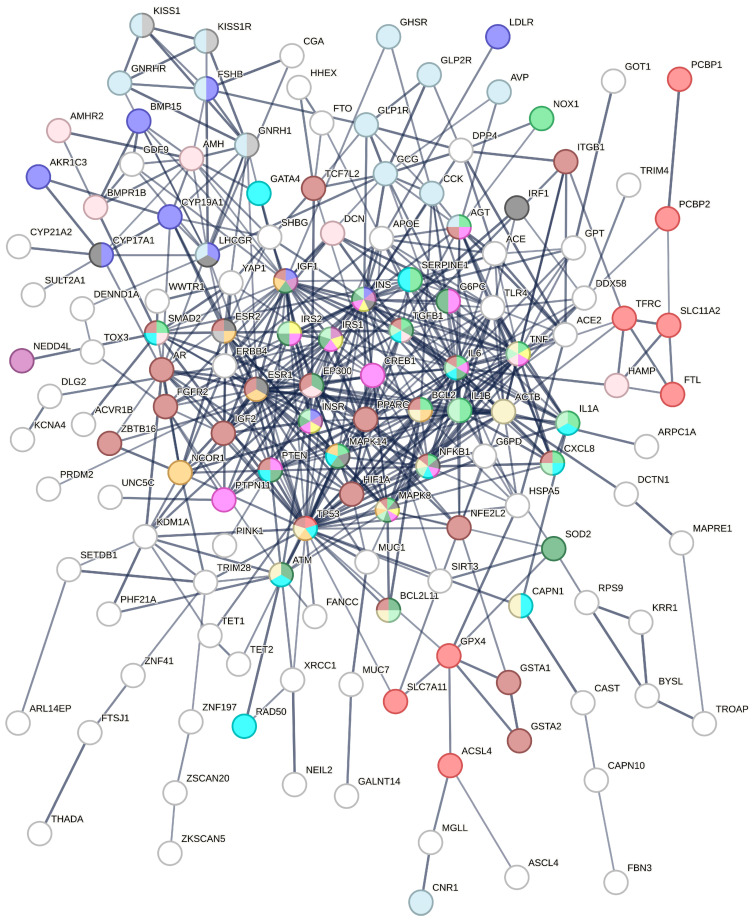
The updated PCOS network: network depicting the associations (connecting lines) of PCOS-relevant genes/gene products (nodes). Over-represented KEGG pathways: FPT (**red**), ovarian steroidogenesis (**blue**), AGE-RAGE signaling pathway in diabetic complications (**light green**), aldosterone-regulated sodium reabsorption (**mauve**), prolactin signaling pathway (**dark gray**), type II diabetes mellitus (**yellow**), insulin resistance (**magenta**), FoxO signaling pathway (**olive green**), TGF-beta signaling pathway (**pastel pink**), cellular senescence (**turquoise**), non-alcoholic fatty liver disease (**pale green**), endocrine resistance (**pale orange**), apoptosis (**light buff**), GnRH secretion (**light gray**), pathways in cancer (**brown**), and neuroactive ligand–receptor interaction (**blue-gray**). The nodes that are not over-represented are marked in white.

Collectively, 82 PCOS-related and 38 FPT–PCOS-associated genes/proteins were retrieved. Moreover, 20 “novel” genes/proteins connecting the “unconnected” input nodes (i.e., not joined to the core PCOS network) were predicted. A total of 140 nodes formed a highly interconnected network (Figure 2), suggesting physical or functional associations within the context of PCOS. The average node degree was calculated to be 7.3. All molecules included in the network are listed in alphabetical order in Table 2. The nodes associated with FPT are marked with purple and the predicted ones in green in Table 2. The major hubs are described in Table 3.

### 3.2. KEGG Analysis Results

**Figure 3:**
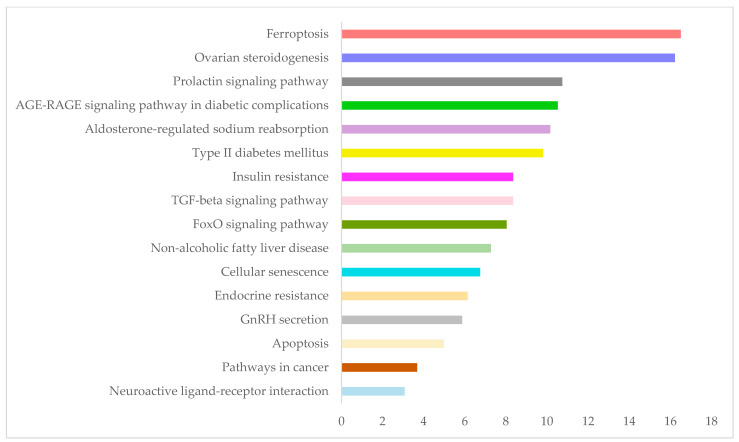
The *x*-axis represents the enrichment ratio of the number of observed genes to the number of expected genes from each KEGG category in the input gene set.

The over-represented KEGG pathways in the PCOS-relevant genes are described in Table 4; the major hubs of the herein presented updated interactome are underlined. Accordingly, in Figure 3, the enrichment analysis results of KEGG pathways are illustrated. The color code adopted in Figure 3 is similar to the one adopted in the pathway highlighted in Figure 2.

### 3.3. FPT Related to the PCOS Molecular Subnetwork

**Figure 4:**
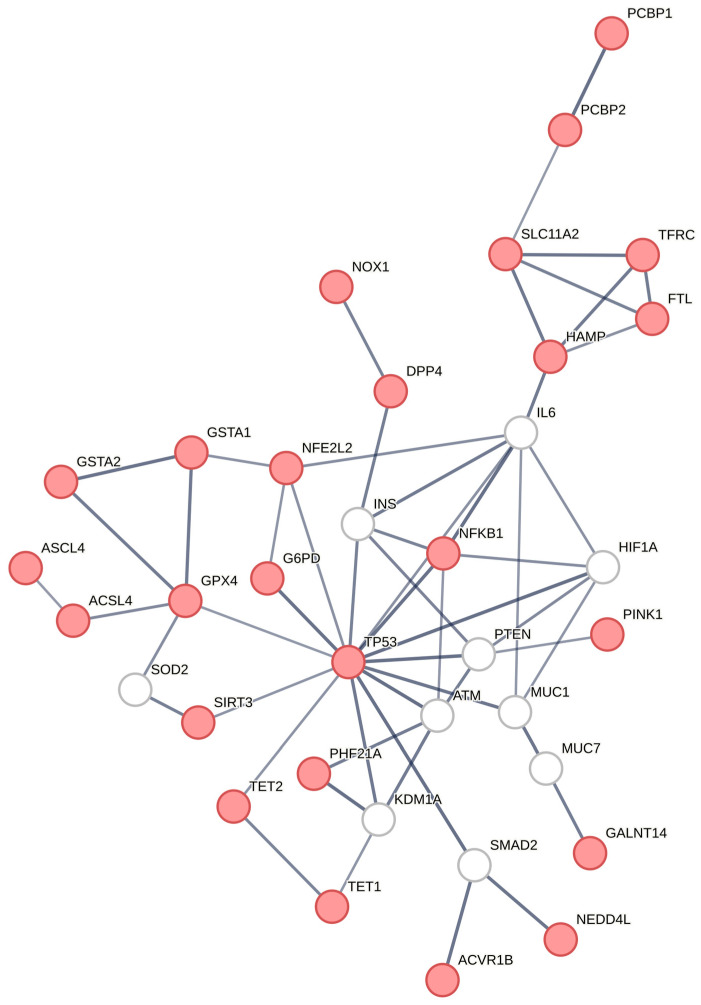
PCOS-related FPT subnetwork; the KEGG FPT enriched pathway nodes are marked in red (as in Figure 3), and the mediating nodes are shown in white.

The herein created PCOS-related FPT subnetwork (Figure 4) includes the FPT-associated molecules reported in the literature (marked in purple in Table 2) plus the KEGG FPT over-represented pathway (marked in red in Figure 3), as well as the mediating nodes, which are shown in white.

## 4. Discussion

The AKR1C3 enzyme reduces androstenedione (A4) to testosterone (T) in the ovaries, adrenal glands, and adipose tissues, where testosterone is mainly produced in women. [5,15].

### 4.1. Pathways

In the present study, we deciphered sixteen over-represented KEGG pathways in the PCOS-relevant genes, described in Table 4 and Figure 3 in color code.

#### Major Hubs

The major hubs of this updated PCOS-specific molecular interactions network (Figure 2) are listed in Table 3, and according to the KEGG pathway analysis, the following can be deduced:

1. TP53 is implicated in cancer, FPT, apoptosis, cellular senescence, and endocrine resistance pathways. In fact, it is the main link between FPT and the syndrome, as it consists of the main hub in both networks.
2. ESR1 is embroiled in prolactin signaling in cancer and endocrine resistance pathways.
3. TNF is involved in non-alcoholic fatty liver, apoptosis, diabetes and its complications (AGE-RAGE signaling), insulin resistance, apoptosis, and TGF beta signaling pathways.
4. INS is included in diabetes, insulin resistance, aldosterone-regulated sodium reabsorption, prolactin signaling, ovarian steroeidogenesis, FoxO signaling, and non-alcoholic fatty liver pathways.
5. TGFB1 is involved in cancer, FoxO, non-alcoholic fatty liver, AGE-RAGE, and TGF beta signaling pathways.
6. EP300 is implicated in cancer, FoxO, and TGF beta signaling pathways.
7. ACTB crosses only the apoptosis pathway.
8. IL6 is included in cancer, FoxO, non-alcoholic fatty liver, AGE-RAGE, insulin resistance, and cellular senescence pathways.
9. IGF1 entangles with endocrine resistance, aldosterone-regulated sodium reabsorption, ovarian steroeidogenesis, cancer, and FoxO pathways.
10. IL1B is embroiled in non-alcoholic fatty liver and AGE-RAGE signaling pathways.
11. PPRAG is implicated in the cancer (but in the literature, in atherosclerosis, obesity, etc.) pathway.

Seven major hubs (EP300, NFKB, ESR1, PPRAG, IGF1, IL6, and TP53) appear to entangle with cancer pathways. Shetty et al. [16] reviewed ten articles studying the risk of developing cancer in those with PCOS, concluding that the controversial evidence published originates in the complexity of the syndrome pathophysiology. Hyperandrogenism, hyperinsulinemia, dyslipidemia, chronic inflammation, and unopposed estrogen action seem to play key roles in this direction. Shetty and colleagues [16] concluded that women with PCOS are at higher risk of developing endometrial cancer but not ovarian or breast cancer. Moreover, a recent meta-analysis showed that women with a family history of PCOS have a lower risk of developing ovarian cancer [17].

### 4.2. Ferroptosis

FPT was described two decades ago as a non-apoptotic cell death mechanism featured by iron-dependent reactive oxygen species (ROS) [18]. Iron is proviso for a variety of cellular functions including homeostasis [19]; thus, FPT seems to be implicated in the pathophysiology of plenty of morbid entities accordingly (i.e., cardiomyopathy, diabetes mellitus, Parkinson’s disease, renal failure, cancer) [20,21]. The FPT–related genes can be classified into drivers, suppressors, markers, inducers, inhibitors, and diseases [22]. The heavy (H) and light (L) chain subunits of ferritin (FTH and FTL, respectively) are responsible for intracellular iron storage and thus are established markers of FPT [23]. Iron ingestion activates NOX1 signaling (through transferrin receptor (TFRC), promoting mitochondrial damage [24,25]. This finding drove Bennett et al. to suggest that folliculogenesis inhibition by TFRC/NOX1 signaling might be a potential drug target of PCOS [26].

The PHD finger protein 21A (PHF21A)-mediating mechanism explaining the FPT involvement in PCOS pathophysiology has not been studied or reported so far [27].

We have created a subnetwork, illustrated in Figure 4, where we reveal all the FPT-related interactions extracted from the KEGG analysis, as well as all those sparse results reported in the literature. In this subnetwork, we untangle the FPT–PCOS-related full interactions network for the first time.

Our interactions molecular network unraveled that the FPT subnetwork connects with the whole network through lysine demethylase 1A (KDM1A) (Figure 2 and Figure 4). KDM1A, in turn, interacts with TP53 and TET1, TRIM28, AR, MUC1, ESR1, and SETDB1 directly (Figure 2). NCOR1 regulates B lymphocyte development, thus determining immunity to health or morbidity directions [28]. This remark ascertains that the roles of PHF21A and NCOR1 merit further elucidation with in vivo or in vitro experiments (bed and bedside research).

It has been shown that TRIM28 is involved in DNA damage repair, although the means are uncertain, possibly by triggering cell cycle arrest [29].

The histone methyltransferase SETDB1, a common ovarian gene, has served as a marker of PCOS efficacy treatment [30].

Mutations in ESR1/2 and enhanced androgenic (AR) activity are established causes of PCOS. Yet, according to Sagvekar and collaborators’ (2022) investigation, epigenetic peripheral DNA methylation changes in CGCs of women with PCOS may arise partly due to intrinsic alterations in the transcriptional regulation of TET1 and DNMT3A [31].

TP53 is the major hub of the FPT subnetwork, apart from the whole PCOS interactome. It is a known regulator of cell cycle metabolism and apoptosis [32,33]. *TP53* mutation influences energy metabolism facets in many ways [34,35]. Although it represents an established cancer marker (suppressor) [33,36], it is implicated in many pathways linking many nodes and enhancing PCOS complexity. In our interactome, it is implicated in insulin resistance, FPT, and prolactin signaling non-alcoholic fatty liver (NAFL) pathways. More importantly, it connects directly with INS—the node that connects metabolic—related genes (KISS1, IGF1, CCK, AGT, AVP, FTO, GCG, PPRAG, SERPINE1, LEP, GRHL, GHSR, cytokines, and interleukins) to reproduction-related ones (AR, GNRH1, SHBG, and LHCGR). Indeed, KEGG pathway analysis proved that INS is implicated in ovarian steroidogenesis, FOXO1 signaling, and aldosterone-regulated sodium reabsorption pathways apart from IR, NAFL, DM2, and prolactin signaling pathways.

Insulin resistance (IR) is an established component of NAFL according to the “second” and the “multiple strike” (lipotoxicity, mitochondrial dysfunction, activation of the inflammatory pathway, and an imbalance of the intestinal microbiota) theories [37]. Accordingly, the literature evidence supports that IR represents a major hazard for NAFL in PCOS as well [38,39].

GOT1 is an established key regulator of glutamate levels, the main excitatory neurotransmitter of the vertebrate central nervous system. It acts as a scavenger of glutamate in brain neuroprotection. It is associated with NAFL and cholocystitis, as well as siderosis (deposition of iron) [6,40].

GPT has been linked to NAFL in children and adolescents [41]. It has been established that androgens may lead to mitochondrial β-oxidation imbalance and de novo lipogenesis through PPARs and can exacerbate liver inflammatory damage by upregulating the expressions of cytokines such as IL-6, TNF-α, and IL-1β [42]. So far, the proposed treatment of NAFL in PCOS includes life style changes, GLP1 receptor agonists, spironolactone, thiazolidinedione (in non-obese people with PCOS), metformin, and nutritional supplements containing 1000 mg omega-3 fatty acids (containing 400 mg of α-linolenic acid) and 400 IU vitamin E [43].

Moreover, HIF1A has also been identified as a mediator of the FPT-activated angiogenesis in sleep apnea [44].

Although (following the Rotterdam criteria) hyperprolactinaemia (HPRL) is an exclusion criterion for PCOS diagnosis, in our interactome, HIF1A has been included in the prolactin pathway. HPRL and PCOS are the major causative factors of anovulation in women. For 70 years, a notion linking these two pathological entities exists, but the mechanism is still unknown (although many hypotheses have been suggested) [45,46]. The recent literature provides evidence that in PCOS, the HPRL is either temporal or macroprolactinemia-related [46].

Oxygen homeostasis is regulated and/or expressed by HIF1A, as it plays a dual role (regulator and transcription factor); ROS/HIF1A promotes oxidative stress, increasing inflammation, whilst inflammation enhances HIF1A expression accelerating oxidative stress, thus promoting FPT [25], resulting in diminished or non-existent mitochondrial cristae and ruptured and contracted outer mitochondrial membranes. Therefore, our analysis unravels a novel mechanism that actually links the two pathophysiologies, contrary to the belief of Delcour et al., who stated that this is a “myth” [46].

ATM is involved in insulin resistance, TGF-beta, and FOXO1 pathways in the constructed interactome.

The FOXO1 pathway has been reported as significantly elevated in the cumulus cells of PCOS women compared to the ones obtained from non-PCOS individuals, through participating in gluconeogenesis, oxidative stress, cell proliferation, and cell apoptosis [47]. It has been associated with obesity as well [6,48,49].

### 4.3. Predictions—Novel Connectors

The predicted novel connectors (20) from our analysis are the following: ACTB, APOE, BYSL, CAPN1, DCN, DDX58, DLG2, ESR1, FTSJ1, KDM1A, MGLL, MUC1/7, PTEN, RPS9, SMAD2, TROAP, ZNF197/41, and ZSCAN20 (Table 2).

Of those, only ESR1 and ACTB are highly connected hubs (Table 3).

ACTB regulates gene transcription and motility, as well as DNA damage repair [50]. It is known to be associated with thrombocytopenia, deafness, and congenital diseases (i.e., congenital blepharoptosis and juvenile dystonia onset) [51]. Through pathway enrichment analysis and hub gene miRNA networks, Heidarzadehpilehrood et al. highlighted ACTB, KRAS, JUNE, PTEN, and MAPK1 as potential therapeutic targets for PCOS treatment [52].

BYSL is linked to ectopic pregnancy, while CAPN1 (to T cell receptor signaling pathway) and DDX58 are associated with immune response [53,54,55].

DCN is a proteoglycan of the extracellular matrix involved in the differentiation of retinal ganglion cells, exocrine gland phenotype, and metabolic homeostasis, and it affects ovarian function in women [56,57].

The FTSJ1 enzyme modifies rRNA [58].

KDM1A coactivates androgen receptor (AR)-dependent transcription by being recruited to AR target genes and mediating the demethylation of H3K9me, a specific tag for epigenetic transcriptional repression. It might be the one of the links between the syndrome manifestation and the epigenetic lifestyle-related effects [59].

MUC1/7 appear to be components of gene expression alterations that potentially contribute to endometrial insufficiency in people with PCOS and lipid metabolism, accordingly [60,61]. RPS9 is a ribosomal gene and SMAD modulates ovarian steroidogenesis; its defect may lead to hyperandrogenism, becoming a potential drug target for PCOS treatment [62].

APOE alleles have been found to be higher in PCOS patients than their non-PCOS counterparts in various clinical trials [63,64,65,66,67], but the parallel increase in cardiovascular risk has been questioned [66]. The APOE increase in PCOS has been associated with neurodegenerative diseases, such as dementia [65,67].

MGLL encodes monoacylglycerol lipase that catalyzes the monoesters of fatty acids, as well as the endocannabinoid 2-arachidonoglycerol [68,69,70]. To our knowledge, this is the first study that depicts its implication in PCOS. In our interactome, it mediates CNR1 (cannabinoid receptor) with the FPT-associated ACSL4.

Although TROAP’s role in PCOS is unknown, our network unraveled that it links BYSL with MAPRE1, the role of which in PCOS is also undetermined but has been correlated to cell functions and autophagosomes in general [62].

Of those, in our wild-type gene interactions network, only JUNE is absent. ACTB and PTEN are predicted nodes, while PTEN is not a hub for the protein–protein, gene–protein, and gene–gene interaction network.

Estrogen receptor (ESR1) ligand mutations have been linked to endocrine resistance, osteoporosis, and breast cancer [71]. It is an emerging predictive biomarker and chemotherapy guide for breast cancer patients [72].

The “novel” ZSCAN20 interacts physically with ZKSCAN5 based on affinity chromatography and co-immunoprecipitation assays. It is also connected through the “novel” interactor ZNF197 to TRIM28. ZSCAN20 has been associated with atrial tachyarrhythmia and angiosarcoma, whereas ZKSCAN5 is a poor prognostic factor of breast cancer; yet, both cancer types are not associated with the syndrome [16,65].

## 5. Conclusions

Our analysis contributed to a deeper insight into the PCOS physiology and untangled the unknown or contested interactions. We identified the FPT-related subnetwork to the PCOS interactions. More importantly, our interactome a) proposed FPT (via HIF1A-ATM) as the link between HPRL and PCOS and b) unraveled the implication of TROAP and MGLL to PCOS. Furthermore, it provided novel perspectives in this complex disease treatment by shedding light on the significance of already proposed drug targets and unraveled new ones, limiting them to KDM1A, TP53, and RPS9; as TP53 is the major hub of the whole network, it should be granted priority.

## Figures and Tables

**Table 1:** Rotterdam criteria for PCOS phenotype distribution.

PCOS Phenotype	Characterization	Clinical Image
A	Classic	HA + OD + PCOM
B	Non-PCOM PCOS	HA + OD
C	Ovulatory PCOS	HA + PCOM
D	Non-hyperandrogenic PCOS	OD + PCOM

HA, hyperandrogenism; OD, ovulatory dysfunction; PCOM, polycystic ovarian morphology.

**Table 2:** Genes/gene products included in the PCOS network.

Symbol	Name
**ACE**	angiotensin I converting enzyme
**ACE2**	angiotensin converting enzyme 2
**ACSL4**	acyl-CoA synthetase long chain family member 4
**ACTB**	actin beta
**ACVR1B**	activin A receptor type 1B
**AGT**	angiotensinogen
**AKR1C3**	aldo-keto reductase family 1 member C3
**AMH**	anti-Mullerian hormone
**AMHR2**	anti-Mullerian hormone receptor type 2
**APOE**	apolipoprotein E
**AR**	androgen receptor
**ARL14EP**	ADP ribosylation factor-like GTPase 14 effector protein
**ARPC1A**	actin-related protein 2/3 complex subunit 1A
**ASCL4**	achaete-scute family bHLH transcription factor 4
**ATM**	ATM serine/threonine kinase
**AVP**	arginine vasopressin
**BCL2**	BCL2 apoptosis regulator
**BCL2L11**	BCL2-like 11
**BMP15**	bone morphogenetic protein 15
**BMPR1B**	bone morphogenetic protein receptor type 1B
**BYSL**	bystin-like
**CAPN1**	calpain 1
**CAPN10**	calpain 10
**CAST**	calpastatin
**CCK**	cholecystokinin
**CGA**	glycoprotein hormones, alpha polypeptide
**CNR1**	cannabinoid receptor 1
**CREB1**	cAMP responsive element binding protein 1
**CXCL8**	C-X-C motif chemokine ligand 8
**CYP17A1**	cytochrome P450 family 17 subfamily A member 1
**CYP19A1**	cytochrome P450 family 19 subfamily A member 1
**CYP21A2**	cytochrome P450 family 21 subfamily A member 2
**DCN**	decorin
**DCTN1**	dynactin subunit 1
**DDX58**	DExD/H-Box Helicase 58
**DENND1A**	DENN domain containing 1A
**DLG2**	disks large MAGUK scaffold protein 2
**DPP4**	dipeptidyl peptidase 4
**EP300**	E1A binding protein p300
**ERBB4**	erb-b2 receptor tyrosine kinase 4
**ESR1**	estrogen receptor 1
**ESR2**	estrogen receptor 2
**FANCC**	FA complementation group C
**FBN3**	fibrillin 3
**FGFR2**	fibroblast growth factor receptor 2
**FSHB**	follicle stimulating hormone subunit beta
**FTL**	ferritin light chain
**FTO**	FTO alpha-ketoglutarate dependent dioxygenase
**FTSJ1**	FtsJ RNA 2’-O-methyltransferase 1
**G6PC**	glucose-6-phosphatase catalytic subunit 1
**G6PD**	glucose-6-phosphate dehydrogenase
**GALNT14**	polypeptide N-acetylgalactosaminyltransferase 14
**GATA4**	GATA binding protein 4
**GCG**	glucagon
**GDF9**	growth differentiation factor 9
**GHSR**	growth hormone secretagogue receptor
**GLP1**	glucagon-like peptide 1 receptor
**GLP2**	glucagon-like peptide 2 receptor
**GNRH1**	gonadotropin releasing hormone 1
**GNRHR**	gonadotropin releasing hormone receptor
**GOT1**	glutamic oxaloacetic transaminase 1
**GPT**	glutamic pyruvic transaminase
**GPX4**	glutathione peroxidase 4
**GSTA1**	glutathione S-transferase alpha 1
**GSTA2**	glutathione S-transferase alpha 2
**HAMP**	hepcidin antimicrobial peptide
**HSPA5**	heat shock protein family A (Hsp70) member 5
**HHEX**	hematopoietically expressed homeobox
**HIF1A**	hypoxia inducible factor 1 subunit alpha
**IGF1**	insulin-like growth factor 1
**IGF2**	insulin-like growth factor 2
**IL1A**	interleukin 1 alpha
**IL1B**	interleukin 1 beta
**IL6**	interleukin 6
**INS**	insulin
**INSR**	insulin receptor
**IRF1**	interferon regulatory factor 1
**IRS1**	insulin receptor substrate 1
**IRS2**	insulin receptor substrate 2
**ITGB1**	integrin subunit beta 1
**KCNA4**	potassium voltage-gated channel subfamily A member 4
**KDM1A**	lysine demethylase 1A
**KISS1**	KiSS-1 metastasis suppressor
**KISS1R**	KISS1 receptor
**KRR1**	KRR1 small subunit processome component homolog
**LDLR**	low-density lipoprotein receptor
**LHCGR**	luteinizing hormone/choriogonadotropin receptor
**MAPK14**	mitogen-activated protein kinase 14
**MAPK**	mitogen-activated protein kinase 8
**MAPRE1**	microtubule-associated protein RP/EB family member 1
**MGLL**	monoglyceride lipase
**MUC1**	mucin 1, cell surface associated
**MUC7**	mucin 7, secreted
**NCOR1**	nuclear receptor corepressor 1
**NEDD4L**	NEDD4-like E3 ubiquitin protein ligase
**NEIL2**	nei-like DNA glycosylase 2
**NFE2L2**	NFE2-like bZIP transcription factor 2
**NFKB1**	nuclear factor kappa B subunit 1
**NOX1**	NADPH oxidase 1
**PCBP1**	poly(rC) binding protein 1
**PCBP2**	poly(rC) binding protein 2
**PHF21A**	PHD finger protein 21A
**PINK1**	PTEN induced kinase 1
**PPARG**	peroxisome proliferator-activated receptor gamma
**PRDM2**	PR/SET domain 2
**PTEN**	phosphatase and tensin homolog
**PTPN11**	protein tyrosine phosphatase non-receptor type 11
**RAD50**	RAD50 double strand break repair protein
**RPS9**	ribosomal protein S9
**SERPINE1**	serpin family E member 1
**SETDB1**	SET domain bifurcated histone lysine methyltransferase 1
**SHBG**	sex hormone binding globulin
**SIRT3**	sirtuin 3
**SLC11A2**	solute carrier family 11 member 2
**SLC7A11**	solute carrier family 7 member 11
**SMAD2**	SMAD family member 2
**SOD2**	superoxide dismutase 2
**SULT2A1**	sulfotransferase family 2A member 1
**TCF7L2**	transcription factor 7-like 2
**TET1**	tet methylcytosine dioxygenase 1
**TET2**	tet methylcytosine dioxygenase 2
**TFRC**	transferrin receptor
**TGFB1**	transforming growth factor beta 1
**THADA**	THADA armadillo repeat containing
**TLR4**	Toll-like receptor 4
**TNF**	tumor necrosis factor
**TOX3**	TOX high-mobility group box family member 3
**TP53**	tumor protein p53
**TRIM28**	tripartite motif containing 28
**TRIM4**	tripartite motif containing 4
**TROAP**	trophinin-associated protein
**UNC5C**	unc-5 netrin receptor C
**WWTR1**	WW domain containing transcription regulator 1
**XRCC1**	X-ray repair cross complementing 1
**YAP1**	Yes1-associated transcriptional regulator
**ZBTB16**	zinc finger and BTB domain containing 16
**ZKSCAN5**	zinc finger with KRAB and SCAN domains 5
**ZNF197**	zinc finger protein 197
**ZNF41**	zinc finger protein 41
**ZSCAN20**	zinc finger and SCAN domain containing 20

FPT–PCOS-associated (in purple);Novel Connectors (in green).

**Table 3:** Major hubs of the PCOS network.

Hub	Connections
TP53	41
INS	34
IL6	33
ESR1	29
IL1B	29
IGF1	27
TNF	26
ACTB	24
EP300	23
NFKB1	21
PPARG	21
TLR4	20

**Table 4:** Over-represented KEGG pathways in the PCOS-relevant genes. The major hubs of the herein presented updated interactome are underlined.

KEGG Patwhay	Genes
AGE-RAGE signaling pathway in diabetic complications	AGT BCL2 CXCL8 IL1A IL1B IL6 MAPK14 MAPK8 NFKB1 NOX1 SERPINE1 SMAD2 TGFB1 TNF
Ovarian steroidogenesis	AKR1C3 BMP15 CYP17A1 CYP19A1 FSHB IGF1 INS INSR LDLR LHCGR
FoxO signaling pathway	ATM BCL2L11 EP300 IGF1 G6PC IL6 INS INSR IRS1 IRS2 MAPK14 MAPK8 PTEN SOD2 TGFB1
Non-alcoholic fatty liver disease	BCL2L11 CXCL8 GPT GOT1 IL1A IL1B IL6 INS INSR IRS1 IRS2 MAPK8 NFKB1 TGFB1 TNF
Ferroptosis	ACSL4 FTL GPX4 PCBP1 PCBP2 SLC11A2 SLC7A11 TFRC TP53
Pathways in cancer	AGT AR BCL2 BCL2L11 CXCL8 EP300 ESR1 ESR2 FGFR2 GSTA1 GSTA2 HIF1A IGF1 IGF2 IL6 ITGB1 MAPK8 NFE2L2 NFKB1 PPARG PTEN SMAD2 TCF7L2 TGFB1 TP53 ZBTB16
Cellular senescence	ATM CAPN1 CXCL8 GATA4 IL1A IL6 MAPK14 NFKB1 PTEN RAD50 SERPINE1 SMAD2 TGFB1 TP53
TGF-beta signaling pathway	AMH AMHR2 BMPR1B DCN EP300 HAMP SMAD2 TGFB1 TNF
Insulin resistance	AGT CREB1 G6PC IL6 INS INSR IRS1 IRS2 MAPK8 NFKB1 PTEN PTPN11 TNF
Prolactin signaling pathway	CYP17A1 ESR1 ESR2 INS IRF1 LHCGR MAPK14 MAPK8 NFKB1 HIF1A
Type II diabetes mellitus	INS INSR IRS1 IRS2 MAPK8 TNF
Endocrine resistance	BCL2 ESR1 ESR2 IGF1 MAPK14 MAPK8 NCOR1 TP53
Apoptosis	ACTB ATM BCL2 BCL2L11 CAPN1 MAPK8 NFKB1 TNF TP53
Neuroactive ligand–receptor interaction	AGT AVP CCK CNR1 FSHB GCG GHSR GLP1R GLP2R GNRH1 GNRHR KISS1 KISS1R LHCGR
Aldosterone-regulated sodium reabsorption	IGF1 INS INSR IRS1 NEDD4L
GnRH secretion	ESR2 GNRH1 KISS1 KISS1R

## Data Availability

All data and analysis methodologies are contained in the manuscript. Any additional data requests can be addressed to the corresponding author.
